# The cauldron has cooled down: a systematic literature review on home advantage in football during the COVID-19 pandemic from a socio-economic and psychological perspective

**DOI:** 10.1007/s11301-021-00254-5

**Published:** 2022-01-04

**Authors:** Michael Christian Leitner, Frank Daumann, Florian Follert, Fabio Richlan

**Affiliations:** 1 Centre for Cognitive Neuroscience (CCNS), University of Salzburg, Hellbrunnerstrasse 34, 5020 Salzburg, Austria; 2 Department of Psychology, University of Salzburg, Hellbrunnerstrasse 34, 5020 Salzburg, Austria; 3 Faculty of Social and Behavioural Sciences, Friedrich-Schiller-University Jena, Seidelstraße 20, 07749 Jena, Germany; 4 Faculty of Management, Seeburg Castle University, Seeburgstraße 8, 5201 Seekirchen, Austria

**Keywords:** Fans, Home advantage, Football, Systematic literature review, COVID-19, D91, L83, Z20, Z29

## Abstract

The phenomenon of home advantage (home bias) is well-analyzed in the scientific literature. But only the COVID-19 pandemic enabled studies on this phenomenon—for the first time in history—on a global scale. Thus, several studies to date examined the effects of empty stadiums by comparing regular matches (with supporters) before the COVID-19 restrictions with so-called ghost games (games without supporters) during the pandemic. To synthesize the existing knowledge and offer an overview regarding the effects of ghost games on home advantage we provide a systematic literature review on this topic. Our findings—based on 26 primary studies—indicate that ghost games have a considerable impact on the phenomenon of home advantage. Deeper analysis further indicates that this effect is based on a reduced “referee bias” and a lack of “emotional support from the ranks”. From a psychological perspective, we argue that our conclusions are highly relevant by emphasizing decision making under pressure and crowd-induced motivation in sports. From a socio-economic perspective, we argue that our findings legitimize a discussion regarding compensation of fans after sporting success as plausible and worth considering. Thus, our results are significant for scientists, sports and team managers, media executives, fan representatives and other persons responsible in the football industry.

**Supplementary Information:**

The online version contains supplementary material available at 10.1007/s11301-021-00254-5.

## Introduction

Football (soccer) is considered the biggest game on this planet and especially the excitement and unpredictability of matches is intriguing for fans around the globe (Schreyer et al. 2018). Fans and spectators are commonly referred to as the “12th man” (Saunders 2020) and constitute an important part of the modern entertainment product “professional football” (Edensor 2015). From a scientific perspective, fans are seen as an (external) determinant in the production of sporting success, and consequently of economic success (Daumann 2019; Dietl et al. 2005;). Thus, football benefits greatly from active spectators (Rudolph et al. 2017), whether through ticket revenues or through the typical stadium atmosphere that gives the product its special marketability (Woratschek et al. 2019). These benefits, however, are also accompanied by (external) costs—such as violence (Di Domizio and Caruso 2015; Dunning et al. 1986; Mause 2020; Singleton et al. 2021)—posing considerable challenges for sporting and political players that regularly have to be borne by the clubs and the general public.

In the present review, we concentrate on the positive contribution of football fans and suggest interpreting fans as important production factors on various levels (Follert et al. 2020). In this context, the influence of fans on the game results mainly in the so-called “home advantage” (Courneya and Carron 1992) (or home bias) which is well-documented in various scientific studies and experiments (e.g., Jamieson 2010; Nevill et al. 2002). The home advantage states that home teams win more than half of the games (excluding draws) when home and away games are evenly distributed in a season (Courneya and Carron 1992). Thus, a corresponding relative advantage of home teams over away teams can be assumed—albeit to varying degrees and based on different explanations (Buraimo et al. 2012; Courneya and Carron 1992; Pollard 2008; Ponzo and Scoppa 2018).

The COVID-19 pandemic led to laboratory-like conditions in stadiums, enabling the scientific evaluation of (missing) fans on various social and sporting aspects during a football match (Bryson et al. 2021). In particular, these so-called “ghost games” (games without supporters) offer the opportunity to specifically investigate the influence of the audience on the behavior and decisions of referees (Dohmen and Sauermann 2016) and on the behavior and motivation of players (Ponzo and Scoppa 2018). Due to the COVID-19 pandemic, games in the major (European) professional football leagues were suspended for the 2019/20 season in mid-March and then resumed in the form of ghost games in May and June 2020. For an overview on leagues’ suspensions and restarts see Kicker (2021) and Tovar (2021).

After screening the broad relevant empirical literature as an outcome of the ghost games during and after the COVID-19 lockdowns, the additional benefit of another empirical study seems limited. For the international scientific community, however, it may be more important to bundle, to synthesize and to clarify the findings from this field in the (presumable) dusk of the pandemic in the fall of 2021. In particular, there are some inconsistencies across studies regarding the reported effects of the ghost games on the home advantage, which can be tracked down to different methodological approaches, inconsistencies across leagues and small or particular samples (see e.g., Benz and Lopez 2021). To this end, we provide a comprehensive overview of all empirically relevant studies conducted so far. We aim to (1) investigate to what extent the home advantage in football has changed during the ghost games of the COVID-19 pandemic and (2) study the empirical explanatory approaches and psychological principles behind the respective findings. It shall be noted, that since there are well-known differences in the magnitude of the home advantage effect depending on the type of sport (e.g., Jamieson 2010), we focus—in order to maintain homogeneity—our systematic literature review exclusively on football.

Our work pursues the following structure: In Sect. 2, we provide a theoretical background where we highlight previous crucial findings concerning home advantages in sports as well as the role of spectators for the production of a typical stadium atmosphere particularly in professional football. We additionally discuss recent studies on home advantage during the COVID-19 pandemic in other major sports in brief. In Sect. 3, we present the results of our systematic literature review and discuss the main results and limitations. In Sect. 4, we illustrate several implications for further research and the sports business industry before we highlight our conclusions in Sect. 5.

## Theoretical background

### Home advantage in professional sports

Following the work by Courneya and Carron (1992), we understand the home advantage as *“the consistent finding that home teams in sport competitions win over 50% of the games played under a balanced home and away schedule”* (p. 13). First empirical evidence for the home advantage in sport competition was provided by Schwartz and Barsky (1977) who analyzed the major leagues in baseball, American football, hockey, and basketball as well as the U.S. college competition in football and basketball. There are various influencing parameters, which Courneya and Carron (1992) systematize as follows:

1. Game location factors (crowd, learning, travel, rules)
2. Critical psychological states (competitors, coaches, officials)
3. Critical behavioral states (competitors, coaches, officials)
4. Performance outcomes (primary, secondary, tertiary)

Game location factors comprise four determinants that can affect the visiting and home team differently. This includes the crowd factor (1.), according to which the home team receives greater support at home than the visiting team. Learning/familiarity factors (2.) assume that the home team is better acquainted with the location and also has the possibility to redesign it at short notice. In football, the watering of the lawn could be an example. Travel factors (3.) means the consideration that visiting teams have to travel to the venue and thus have to take on inconveniences to which the home team is not exposed. Finally, under rule factors (4.), components of the set of rules are subsumed that provide for favoring the home team. An example is the last line change in ice hockey (Carron et al. 2005).

These four game location factors directly affect the critical psychological, behavioral and performance states of the three relevant groups (competitors, coaches and officials). Coruneya and Carron (1992) differentiate here between three levels: The primary level describes the basic level of performance (such as the distances covered in football or the number of sprints). The second level describes the intermediate or scoring aspect of the performance (e.g. the number of goals scored). The third level records the result measure (in football this would be the number of points that a team scores in a game).^1^

While, for example, travel efforts for teams stayed identical during the COVID-19 pandemic,^2^ crowd influence is of particular interest when analyzing the effects of the COVID-19 pandemic on sporting events. Relevant parameters here are crowd size or density (e.g., Dowie 1982; Goumas 2012; Pollard 1986). Structure and properties of the stadium—the basis for the production of atmosphere by fans and spectators—is also considered as a significantly influencing factor in previous studies (Unkelbach and Memmert 2010).

### The role of fans in producing atmosphere and sporting success

In addition to their classic function as demanders and consumers of the entertainment service produced, fans also play a role in other parts of the value creation process. Woratschek et al. (2014) emphasize the value contribution for the production by, e.g., creating banners or preparing creative choreographies or supporting their team with battle chants. In this context, the ghost games of the COVID-19 pandemic particularly illustrate the high relevance of fans for atmospheric and emotional sports entertainment.

The atmosphere within a stadium can be an important and beneficial factor for football fans (Flatau and Emrich 2016) and—although this finding may be intuitive—it is important to state that the typical stadium atmosphere is an original output of the fans themselves. In this context, we further follow the argument of Ednesor (2015) who states that *“atmosphere is ( …) a co-production that involves players, match organisers, and fans”* (p. 82). Both sides contribute to the production of the game atmosphere through mutual interaction. In Mauss' (1966) sense, the relation can even be interpreted as an exchange. From the player’s perspective, the stadium represents a stage, similar to that of an actor in a theater. Basically, it is about the self-presentation of the players in public. But the player needs the spectator, the big stage, to present himself and his performance (Gebauer 1972; Goffman 1959; Horky 2020). The fans cheer on the team and in return the team fights on the pitch, so that the marketable entertainment service "football" is produced as a result. This makes the unique situation of the COVID-19 pandemic especially interesting as there are no fans in the stadium, creating a highly unusual and mostly first-time situation for many players and clubs. Furthermore, there is the commonly known circumstance that supporters are considered as the "12th man" who influence the outcome of the game in favor of their team (Edensor 2015), making ghost games especially intriguing for socio-economic and psychological analysis. Indeed, analyses of football matches played behind closed doors in the pre-COVID-19 era evidenced the impact of the fans on referee decisions (Pettersson-Lidbom and Priks 2010; Reade et al. 2020a, b).

### COVID-19 and its influence on non-football team sports

The opportunities presented by the COVID-19 pandemic to study home advantage have been used for different team sports. McHill and Chinoy (2020) used the COVID-19-related changes of the game settings to analyze mainly the effect of traveling on the team performance. The NBA ‘s 2019/2020 season was continued after an approximately six-month break with 22 teams in Orlando ("the bubble"). In the first games, spectators were completely excluded. McHill and Chinoy (2020) showed that traveling across time zones has a negative effect on winning percentage, team shooting accuracy, and turnover percentage. Furthermore, traveling in general has a negative effect on offensive rebounding and increases the number of points the opposing (home) team scores.

Higgs and Stavness (2021) showed on the basis of a Bayesian multilevel regression model that in the NHL and the NBA the home advantage decreased due to the exclusion of spectators, while in the MLB and the NFL hardly any effects were found. These results concerning the NBA were confirmed by Leota et al. (2021). In their study, in which they used mixed models, they showed that in games with crowds, the home team won 58.65% of the games. In games without crowds, the home team only won 50.60% of the games.

Guérette et al. (2021) examining the influence of crowds on the number of penalties called by referees in the NHL, came to similar conclusions as Higgs and Stavness (2021) with regard to the NHL. They showed that in the presence of crowds, home teams are favored by referees concerning penalties. If games take place without crowds, the number of penalties awarded does not differ significantly between away teams and home teams.

The results of Higgs and Stavness (2021) for the MLB are supported by Losak and Sabel ‘s (2021) research. According to their analysis, the presence of the crowd is not a driver of the home field advantage. Likewise, according to their research, travel fatigue does not seem to have any influence on home advantage. Zimmer et al. (2021) come to comparable results in their study of the MLB; although there might be differences between star-batters and nonstar-batters (Jane 2021).

Fioravanti et al. (2021) examined 1027 rugby union matches from 11 tournaments in 10 countries. They came to the conclusion that the exclusion of spectators has the effect that home teams win less matches and that their points difference decreases.

## Method

### Literature identification process

A large number of studies on ghost games have been conducted and published during the COVID-19 pandemic. In order to provide a comprehensive overview on these studies’ data, methods and findings, we decided to analyze the potential effects of ghost games on football with the help of a systematic literature review.^3^ This approach is frequently used in psychology, economics and management research to present the state of the art in a certain field of research (Fisch and Block 2018; Frank and Hatak 2014; Webster and Watson 2002).^4^

On April 28th, 2021 we used the databases *EBSCO-Host*, *EconStor*, *SURF*, *Emeraldinsight*, *JSTOR*, *Sciencedirect*, *Springerlink* and *Google Scholar* to search with the terms *COVID-19, football, soccer, behind closed doors, ghost game,* and *home advantage* for related studies. These databases were selected because we assume that the relevant topic is comprehensively covered due to the breadth of these databases. We used the following search strategies:

1. 'COVID-19' AND 'football' AND 'ghost game'
2. 'COVID-19' AND 'soccer' AND 'ghost game'
3. 'COVID-19' AND 'football' AND 'behind closed doors'
4. 'COVID-19' AND 'soccer' AND 'behind closed doors'
5. 'COVID-19' AND 'football' AND 'home advantage'
6. 'COVID-19' AND 'soccer' AND 'home advantage'
7. 'COVID-19' AND 'home advantage'

The keywords mentioned were only searched for in the titles of the articles. Newspaper articles and comments were excluded,^5^ so that in the end only articles from journals and working papers remained. Papers that were not relevant for the scope of the present work (e.g., investigating sport-medical consequences of ghost games) were also excluded.^6^ This was particularly the case with the EconStor and Springerlink databases. Against this background, Table 1 shows the following results that were obtained.

**Table 1:** Results of the database analysis (April 28, 2021). The number of contributions that are not relevant is given in parenthesis

Query Database	COVID-19, football, ghost games	COVID-19, soccer, ghost games	COVID-19, football, behind closed doors	COVID-19, soccer, behind closed doors	COVID-19, football, home advantage	COVID-19, soccer, home advantage	COVID-19, home advantage
EBSCO_HOST	1	1	0	0	0	1	2
EconStor	2	2 (1)	1	1	3	3 (1)	11 (6)
Emeraldinsight	0	0	0	0	0	0	0
Google Scholar	0	1	0	0	5	2	13 (1)
JSTOR	0	0	0	0	0	0	0
Sciencedirect	0	0	0	0	0	0	0
Springerlink	0	0	7(7)	2 (2)	0	0	0
SURF	0	0	0	0	0	0	0

Additionally, we manually worked through the references of these studies in order to find manuscripts that had been overlooked by our initial search. Here, one study provided only descriptive data and the focus was not on home advantage, and one paper was published in a journal whose doi was not accessible. After we removed duplicates, originally 16 studies (10 peer-reviewed) formed our sample to be analyzed. In this context, the Econstor database in particular showed that sometimes several hits related to the same article. Especially the work from Reade et al. (2021) provided valuable information to fully complete our dataset. After the review process—which was finalized in December 2021—26 studies (20 peer-reviewed) were finally included into the present analysis.^7^ Note that all studies included are marked with a hashtag (#) in the references section.

### Systematic literature review characteristics

Besides basic properties and information (year, authorship, journal, peer-review status, etc.) the following characteristics of every study were extracted and documented in detail: number of reported countries, number of analyzed leagues, method of comparison (e.g., pooled and/or not pooled leagues), number and properties of analyzed factors (e.g., goals, cards, fouls, etc.), number and properties of evaluation methods and statistical approaches, results and (central) conclusion.

In order to quantify these factors, we created an individual “Spectrum score” for every included study—used as a proxy that allows a comparison in terms of breadth and depth of analysis, methodological design, statistics used and factors included. In this way we assessed the studies’ crucial components: “Leagues”, “Leagues comparison”, “Seasons”, “Main factors”, “Side factors” and “Evaluation”. To achieve a final score for each work the studies were analyzed individually for similarities and differences in their analytical approach, and weightings were used respectively (also see supplementary material) to provide a balanced assessment of the included characteristics. The weighting was based on calculated mean values across all studies. This approach prevented individual study characteristics from having a biasing influence on the respective overall picture, represented in the concluding “Spectrum score”, which calculation is based on the following factors:

A. *Leagues* We divided all leagues from AFC, CONCACAF, CONMEBOL and UEFA, depending on their official, individual league scores into different tiers ranging from 1 to 5. The highest tier 1 includes England, Spain, Italy, and Germany. Including first leagues from this tier into a study was awarded with 0.75 points per league, second leagues with 0.5 points, third leagues with 0.25 points and all leagues below with 0.1 points. When all Top 4 Leagues were included 5 bonus points were added on top. We argue based on work from Agnew and Carron (1994), Fischer and Haucap (2021), Goumas (2012) and Unkelbach and Memmert (2010) that the difference between regular audience and ghost games has an impact on the effect size and the influence from the ranks. This difference is larger in higher and thus more popular leagues than in lower and less popular leagues and especially among the top clubs and top leagues of this world who emotionalize fans across the globe. The final score resulted in the “Combined leagues subscore”. See also the supplementary material for a detailed description of leagues and their respective weighting.
B. *League comparison* 0 points were awarded to studies calculating with single leagues (“not pooled”—calculating the change of home advantage for single leagues and reporting single results) *or* a group of leagues (“pooled”—calculating the change of home advantage for a group of leagues and reporting an overall result), 2.5 points were awarded to studies calculating with “not pooled” and “pooled data”.
C. *Seasons* The number of included seasons since 2015 was weighted with a score of 0.5, with a score of 0.25 for data from season 2010 to 2014 and 0.1 when data from seasons before the year 2010 was included into the study. We argue that, on the one hand, including too many seasons introduces potentially confounding trends into the respective studies. On the other hand, studies based on a small number of seasons potentially yield less robust statistical results. Thus, with the weightings we create a compromise between temporal proximity and thus better comparability to the critical season(s) of ghost games and studies with a large number of seasons, potentially delivering a robust basis for statistical analysis.
D. *Main factors & Side factors* After analyzing every study, we identified and extracted 4 main factors on home advantage (win ratio *or* points, goals, cards and (regular) attendance). Following the same logic, we further found several side factors on home advantage (e.g., shots, fouls, corners, possession, etc.). These findings are largely consistent with previous theories and concepts on home advantage, which were presented in Chapters 1 and 2 of this study. We awarded 1 point for every main factor and 0.25 points for every side factor included into the study, culminating into an “Analyzed factors subscore”.
E. *Evaluation* We awarded 2.5 points if the findings regarding the effects of home advantage in ghost games were explicitly evaluated and assessed with more than one statistical method or (complementary) approaches to additionally validate the conclusions in the respective study.

The sum of the individual values from the list from A to E forms the "Spectrum score" and thus reflects a concise and objective comparative value—based on included factors and methods—between the different studies.

Besides calculating an individual “Spectrum score” for every study we additionally set another individual value—the “Conclusion score”—representing the central conclusion of each study in terms of the magnitude of the impact of ghost games on home advantage. In order to set this score, we screened every paper for crucial text passages (usually found in the discussion or conclusion section of the manuscript) indicating the authors' verdict on the magnitude of the home advantage in ghost games, based on their findings. A 7-point non-directed likert scale (ranging from (1) “strongly increased home advantage in ghost games” to (4) “no change in home advantage in ghost games” and (7) “strongly reduced home advantage in ghost games”) was used to represent the studies’ conclusion as precisely as possible.

Summing up, we assigned two values to every included study, the “Spectrum score” and the “Conclusion score”. While the "Spectrum score" represents a central statement regarding the depth, breadth, and empirical amplitude of the respective study, the "Conclusion score" reflects the central statement of the authors regarding the influence of ghost games on the home advantage.

## Results

**Fig. 1:**
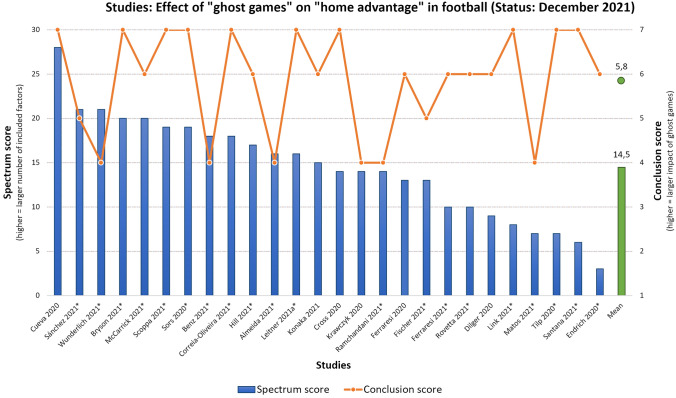
Results illustrating the individual “Spectrum score” (left y-axis and blue bars) with a mean of 14.5 (SD = 5.8)—illustrated by the green bar—and the individual “Conclusion score” (right y-axis and orange line) with a mean of 5.8 (SD = 1.2)—illustrated by the green dot—of all 26 included studies (x-axis) investigating the effect of ghost games on home advantage (HA) in football. Peer-reviewed studies are marked with an asterisk

Analyzing all included 26 studies (20 peer-reviewed), the “Spectrum score” mean value is 14.5 (*SD* = 5.8) and the “Conclusion score” is 5.8 (*SD* = 1.2), as illustrated in Fig. 1. The “Conclusion score” on the right y-axis ranges from 1 to 7, representing the following: 1: strongly increased HA/2: increased HA/3: slightly increased HA/4: no change in HA/5: slightly decreased HA/6: decreased HA/7: strongly decreased HA. The “Spectrum score” on the left y-axis represents a value formed by different subscales (see previous section for details), allowing a quantified comparison between the studies included regarding breadth of analysis, methodological design, statistics used and factors included.

**Fig. 2:**
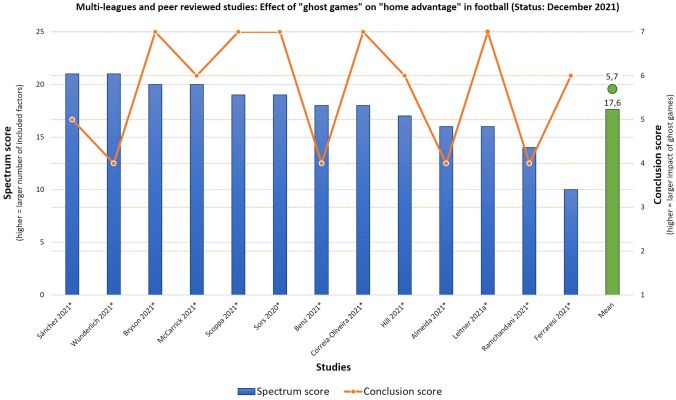
Results illustrating the individual “Spectrum score” (left y-axis and blue bars) with a mean of 17.6 (SD = 3.1)—illustrated by the green bar—and the individual “Conclusion score” (right y-axis and orange line) with a mean of 5.7 (SD = 1.3)—illustrated by the green dot—of 13 both peer-reviewed and multi-leagues studies (x-axis) investigating the effect of ghost games on home advantage (HA) in football. Peer-reviewed studies are marked with an asterisk

When applying more strict methodological and empirical standards to the studies—that is only including peer-reviewed studies and studies investigating more than one league (Top 4 European leagues included)—13 studies with a “Spectrum score” mean of 17.6 (*SD* = 3.1) and “Conclusion score” mean of 5.7 (*SD* = 1.3) remain, as illustrated in Fig. 2.

Based on these findings, we conclude the following: According to current empirical studies, the role of fans seems to be significant for the outcome of matches in professional football. The results further indicate that the home advantage indeed decreases considerably during ghost games (see mean conclusion score in Figs. 1 and 2). There is not a single study that found an *increased* home advantage in ghost games (see Fig. 1): six studies conclude “no change in home advantage”, two studies conclude a “slightly reduced home advantage”, eight studies conclude a “reduced home advantage” and ten studies conclude a “strongly reduced home advantage” in ghost games. When analyzing only peer-reviewed and studies that included the top four leagues (England, Spain, Italy, Germany), the overall conclusion distribution is similar (see Fig. 2): four studies conclude “no change in home advantage”, one study concludes a “slightly reduced home advantage”, three studies conclude a “reduced home advantage” and five studies conclude a “strongly reduced home advantage” in “ghost games”. In this respect and as illustrated in Table 2, the studies’ overall conclusions—based on the respective individual results—indicate that fans have indeed a significant impact on home advantage in professional football.

**Table 2:** *Listing of key findings from the included studies on the relationship between ghost games and home advantage*

Study (first author, year, peer-reviewed(*))	Main conclusion (findings: explanation)
Cueva (2020)	**Strongly reduced home advantage**: Home advantaged dropped by around one half and gap in referees calls between home and away teams disappeared in ghost games *"[…] the home advantage dropped by around one half. The effect of the lockdowns is even more dramatic when it comes to referee calls. While referees made consistently more calls against away teams than against home teams before the lockdowns, this gap completely disappears after the lockdowns."* (p. 3)
Sánchez (2021)*	**Slightly reduced home advantage**: Except for the German and the Spanish top leagues there are no significant differences in wins, points and goals during ghost games *"The results show that there are no significant differences between playing with or without a crowd, except in the German and Spanish top leagues. Even so, there is a tendency in most competitions to play worse at home and better away from home when there are no spectators."* (p. 152)
Wunderlich (2021)*	**No change in home advantage**: Decline of home advantage during ghost games in terms of (reduced) sanctioning of away teams (fouls, yellow and red cards) and (decreased) match dominance of home teams (shots and shots on target) but no significantly decreased home advantage in terms of results *"The present data is evidence that in absence of spectators the increased sanctioning of away teams disappears, the match dominance of home teams remains, but is decreased and the home advantage itself decreases, yet insignificantly."* (p. 12)
Bryson (2021)*	**Strongly reduced home advantage**: Reduced social pressure leads to less yellow cards for away teams *"We find large and statistically significant effects on the number of yellow cards issued by referees. Without a crowd, fewer cards were awarded to the away teams, reducing home advantage. These results have implications for the influence of social pressure and crowds on the neutrality of decisions."* (p. 1)
McCarrick (2021)*	**Reduced home advantage**: Home team performance is significantly decreased in ghost games and referees awarded significantly more fouls and yellow cards against the away teams, which are key elements of the home advantage *"We find points per game, goals per game and team dominance […] were all significantly reduced in the home teams compared to the away teams […] teams won on average 0.39 points per game more at home than away, but this HA was almost halved in the period without the audience; such that the teams won only 0.22 points more at home than away. So, while the HA is present in games played without fans, its impact is reduced by nearly 50% relative to games where fans are present."* (p. 8)
Scoppa (2021)*	**Strongly reduced home advantage**: Home team performance (points, goals, shots, shots on target, corner kicks) deteriorates while away team performance improves in ghost games *“We find considerable effects of the pressure from the crowd: while with the support of the crowd a considerable home advantage emerges in various measures of performance (points, goals, shots, *etc*.), this advantage is almost halved when matches are played behind closed doors. Similar effects are found for the behavior of referees: decisions of fouls, yellow cards, red cards and penalties that tend to favor home teams in normal matches, are much more balanced without the crowd pressing on referees.”* (p. 1)
Sors (2020)*	**Strongly reduced home advantage**: Results indicate that crowd noise has a relevant role for referee decisions (and so for the home advantage) as it was found that both the referee bias and the home advantage decreased in ghost games *"The results bring further support to the claim that, among all the factors contributing to home advantage and referee bias, crowd noise has a relevant role. Thus, spectators can significantly contribute to determine the dynamics and the outcomes of professional football matches."* (p. 1)
Benz (2021)*	**No change in home advantage**: Mixed findings indicate that changes of home advantage in ghost games is league dependent, indicating a complex causal mechanism *"In some leagues, evidence is overwhelming that HA declined for both yellow cards and goals. Alternatively, other leagues suggest the opposite, with some evidence that HA increased."* (p. 20)
Correia-Oliveira (2021)*	**Strongly reduced home advantage**: Overall home advantage was significantly decreased *"Our results suggest that the break due to the COVID-19 pandemic during the 2019/2020 season was detrimental to most teams playing at home without crowd support, with a strong relationship between home advantage and team quality."* (p. 6)
Hill (2021)*	**Reduced home advantage**: In ghost games, performance of home teams (goals scored) decreased and referees decided less favorable for the home team *"We conclude that the home field advantage may indeed be lost when spectators are absent. However, in future studies, more detailed behavioral analyses are needed to determine the robustness and the behavioral determinants of this phenomenon across leagues and countries."* (p. 1)
Almeida (2021)*	**No change in home advantage**: Overall, the home advantage did not decrease considerably (points won) in European leagues in ghost games, however it depends from league to league *"Overall, the HA did not significantly decrease in European leagues (from 16.4% to 11.6%; trivial effect size [ES]); however, a one-sample t-test revealed that the HA after the COVID-19 break was significantly greater than 0% (small ES). While the HA completely disappeared in the Bundesliga (large ES), its effects remained stable in La Liga (small ES), Premier League and Primeira Liga (trivial ES), and even increased in Serie A (medium ES) after the return. Home teams’ performances in these leagues were influenced to different extents by the COVID-19 situation, especially by playing behind closed doors."* (p. 693)
Leitner (2021a)*	**Strongly reduced home advantage**: Referees perceived less social pressure from the home crowd in ghost games—resulting in more yellow cards for the home teams, regardless from the course of the game—leading to the dissolvement of the home advantage effect *“There are two main findings. First, home teams were booked significantly more often with yellow cards for committing fouls in ghost games. Most importantly, this effect was independent of the course of the games. In contrast, bookings for other reasons (criticism and unfair sportsmanship) changed similarly for both home and away teams in ghost games. Second, the overall home performance and home advantage effect in the respective elite leagues–identified in the respective matches of the regular 2018/19 season–vanished in the ghost games of the 2019/20 season.”* (p. 1)
Konaka (2021)	**Reduced home advantage**: Overall, home teams win less games in ghost games, but effect differs between leagues *"More simply, the home advantage became smaller when the games were conducted behind closed doors"* (p. 9)
Cross (2020)	**Strongly reduced home advantage**: Ghost games reduce the chance of home wins due to less scored goals *"We find that the absence of fans leads to 57% decrease in home field advantage as measured by home minus away goals, with the estimated home effect decreasing from 0.387 to 0.167 goals per game. The absence of fans leads to a 68% decrease in home minus away expected goals, indicating that these changes in home field advantage are not driven by better or worse finishing but are instead indicative of changes in chance creation."* (p. 14)
Krawczyk (2020)	**No change in home advantage**: The effect of reduced home field advantage during the COVID-19 pandemic in the top four European football leagues seems to be a singularity that can only be found in the German Bundesliga *"[…] crowds seem to play a limited role in the emergence of home-field advantage in soccer. Indeed, there is some effect in Germany only. We do not have a definite answer why the Bundesliga is special. A sceptic’s answer is that this is a random blip in the data, with the number of games in each specific league being relatively low."* (p. 8)
Ramchandani (2021*)	**No change in home advantage**: Results indicate no strong evidence to support the existence of the purported "twelfth man" effect in football *"The Italian Serie A and the German Bundesliga were the only leagues where any evidence of a significant decline in inter-season HA (between 2018/19 and 2019/2020) or intra-season HA (between fixtures with and without crowds in 2019/20) was found. Overall, there is insufficient evidence to generalize that the absence of crowds affects HA in football."* (p. 1)
Ferraresi (2020)	**Reduced home advantage**: Ghost games led to a drop in winning points for home teams and halved the home advantage *"We find that the performance of the home team is halved when stadiums are empty, with this effect being more marked for teams whose attendance rate was very high and for those that do not have international experience."* (p. 1)
Fischer (2021)*	**Slightly reduced home advantage**: Decrease of home advantage in ghost games is best explained by the low occupancy rate in the stadia, the effect is less dramatic for teams with low occupancy rates in general *"We find that there is a reduced home advantage in the first [German] division, whereas no change is observed in the second and third divisions […] Hence, the decrease in occupancy to zero at the ghost games has been less dramatic for teams that have been used to low occupancy rates. We cannot find strong evidence for a change in referee behavior or teams’ tactics as main impact channels of occupancy rates on the home advantage. Hence, we argue that psychological reasons are of higher importance."* (p. 1)
Ferraresi (2021)*	**Reduced home advantage**: Home teams miss more penalties when played behind closed doors, especially when attendance was high before lockdown; away teams are less likely to miss penalties behind closed doors, especially when attendance was high before lockdown *"[…] social environment affects the performance of individuals. […] in the absence of audience, away teams are less likely to choke on a penalty kick, especially in stadiums that before the Covid-19 outbreak used to be very crowded. These results are consistent with recent findings that suggest that football team performances are negatively affected by the forced absence of friendly audiences […]. What all of this seems to indicate is that both supportive audience and the size of the support play a key role for success of skill tasks."* (p. 4)
Rovetta (2021)*	**Reduced home advantage**: Results indicate statistical evidence supporting the crowd’s impact on sports and refereeing performance in Serie A *"During the anti-COVID-19 restrictive measures […] a net reduction in the points collected by the teams in home matches was detected. […] In addition, the number of penalties awarded against home teams has increased significantly, approaching the ideal 50%. Since there are valid psychological reasons in the literature to support the crowd’s impact on sports and refereeing performance, it is plausible that our findings are causally related to the absence of cheering."* (p. 7)
Dilger (2020)	**Reduced home advantage**: In ghost games, the referee bias disappears explaining the decrease of home advantage, while no changes in performance can be observed *"Comparing these [ghost] games with the regular ones between the same teams before, we find that the normal advantage for the home team disappears. One reason for this is the disappearances of the home bias of the referees whereas changes in the sportive performance of the teams seem to be irrelevant in this regard."* (p. 1)
Link (2021)*	**Strongly reduced home advantage**: Empty stadiums have reduced home advantage and decreased referee bias *"The absence of crowds has erased home advantage in the Bundesliga, reduced home advantage in Bundesliga 2 regarding performance level and increased the neutrality of refereeing decisions when giving yellow cards."* (p. 10)
Matos (2021)*	**No change in home advantage**: Analysis of ghost games in the Portuguese league shows no considerable change in home advantage but statistical methodology / approach plays a considerable role when estimating this effect *"Overall, despite what might be expectable from recent findings, the lack of an audience in the last 10 rounds of Portuguese Football League 2019–2020 season, due to COVID-19 pandemic, did not affect home advantage."* (p. 1)
Tilp (2020*)	**Strongly reduced home advantage**: Analysis of ghost games in the German league shows a considerable change in home advantage *"[…] the Covid-19 lock-down led to a home disadvantage. One reason for this surprising result could be that the home team is missing an important familiar aspect when playing in their empty stadium without social support from their home audience. Furthermore, both teams know about the HA thus the away team could be more motivated in this unusual situation."* (p. 1)
Santana (2021)*	**Strongly reduced home advantage**: Some game and performance indices changed considerably (pass accuracy and fouls committed) while other factors decreased for home teams (sprints) and overall, home advantage decreased significantly in ghost games *“[…] the two-month break due to COVID-19 world pandemic in the Bundesliga changed some aspects of the game, such as sprints, fouls and moments when goals were scored. Although, a high number of match variables were slightly favorable to home teams, their home advantage was lost.”* (p. 5)
Endrich (2020)*	**Reduced home advantage**: Referees punish home teams equally as away teams during ghost games, which could be caused by the missing of pressure from the stands and reducing the home bias *"We find that pre-Covid19 referees gave fewer fouls and yellow cards for the home team relative to the away team. These differences in fouls and cards changed during the ghost matches so that home teams were treated less favorably than before. This effect is concentrated in matches where support for the away team is particularly weak. The results provide evidence for a home bias in referee decisions through social pressure."* (p. 1)

**Fig. 3:**
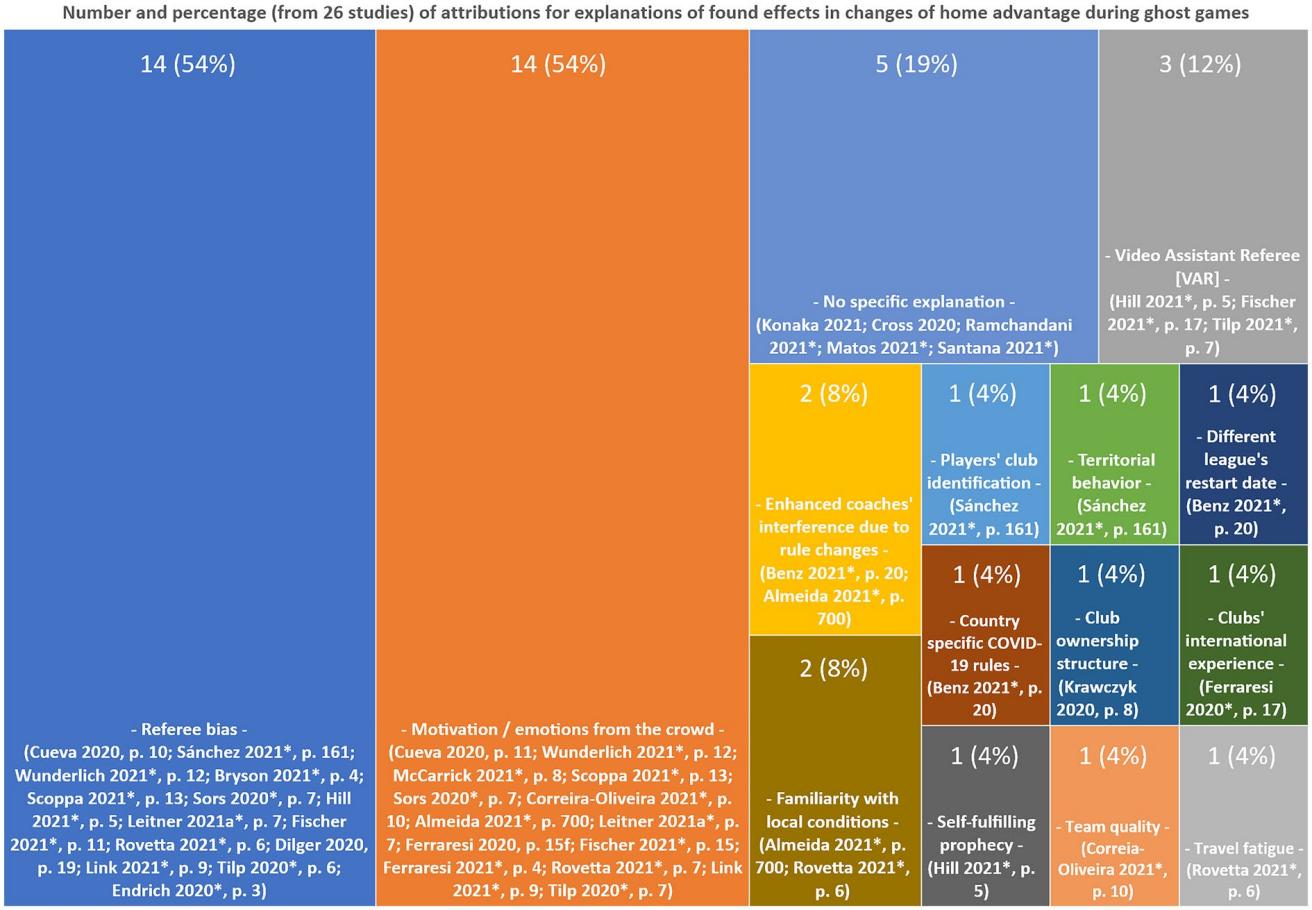
Results illustrate the number of attributions for changes of home advantage during ghost games from the 26 included studies. References to the studies and page numbers—where the respective attributions can be found—are listed at the bottom of each box. Peer-reviewed studies are marked with an asterisk

Furthermore, we analyzed the explanations and reasons given in each study for the respective found effects concerning the home advantage in ghost games (see Fig. 3). We discovered that—from 26 total studies—in 14 studies the “Referee bias”, in 14 studies “Motivation / emotions from the crowd”, in 5 studies “No specific explanation”, in 3 studies the “Video assistant referee (VAR)”, in 2 studies “Enhanced coaches’ interference due to rule changes”, and in 2 studies “Familiarity with local conditions” were explicitly mentioned as reasons for (more or less large) effects in the home advantage during the ghost games of the COVID-19 pandemic. "Players' club identification", "Territorial behavior", "Different league's restart date", "Country specific COVID-19 rules", "Club ownership structure", "Clubs' international experience", "Self-fulfilling prophecy", "Team quality" and "Travel fatigue" were each mentioned in one study as one of the main reasons. It shall be noted that we scanned the studies for explicit explanations for found effects in the context of each study result. This means that mere enumerations of possible explanations—based on previous or other studies—did not qualify as a commitment to one or more explanatory models and thus are not reported.

## Discussion

### Referee bias and the importance of the crowd

Based on the results from our systematic literature review we state the following main findings:

1. A majority of the present studies finds a significant reduction of the home advantage effect during the ghost games of the COVID-19 pandemic
2. This effect holds true in a larger context when single league and not peer-reviewed studies are excluded
3. The two main reasons for this examined effect are the so called “referee bias” and emerging “motivations and/or emotions” from the crowd

With regard to existing explanatory approaches in the literature, theories emerging from social pressure and conformity seem to distill as an empirically conclusive explanatory model for the found effect of decreased home advantage in ghost games. Most studies suggest that a relationship between spectator absence and home advantage can be inferred via the influence on the referee(s) and from a missing effect on players’ emotions, originating from the (home) crowd. Although a less emotional behavior (e.g., Webb 2020), especially a lower level of aggression towards referees, may be desirable from a sporting perspective, it seems to come at the expense of the home advantage and is reflected in a lower sporting success of the home team. The extent of decrease of the home advantage seems to depend on different factors. For example, Fischer and Haucap (2021) analyze matches from the first, second and third league in Germany. Surprisingly, with respect to COVID-19 ghost games, they find a significant decrease in home advantage only for the first Bundesliga when matches are played excluding spectators, possibly due to the larger delta between matches with spectators and ghost games in the first Bundesliga. Furthermore, it is remarkable that a stadiums’ atmospheric conditions seem to significantly depend on its properties (e.g., number of standing places, distance to the field) (Unkelbach and Memmert 2010). If one considers that stadiums are mostly occupied by fans who actively produce atmosphere by singing and cheering for the home team, it can be assumed that the quality of the spectators in terms of their input is relevant for the teams’ success. In this context the power of fans is also becoming increasingly important regarding upcoming reforms in European soccer. A possible European Super League has been discussed for years (e.g., Follert 2019; Follert and Daumann 2021; Follert and Emrich 2020; Littkemann, Geyer and Schmitz, 2021), and time and again the preferences of large parts of the fan scene have been neglected. In the light of our findings from the ghost games of the COVID-19 pandemic, the importance of fans becomes clear not only as a production factor in an economic sense, but also as a significant variable from a sport-psychological perspective.

### Implications for football clubs and organizers

The question now arises as to what implications the empirical evidence might have regarding the club’s governance. In the sports economics literature, it has been discussed for several years to explicitly consider fans in the club's objective function ("fan welfare maximization", e.g., Madden 2012; Madden and Robinson 2012). Although this demand may be criticized with reference to the importance of ownership in a market economy system and the advantages of having an investor as “sugar daddy” (Franck 2010; Richau et al. 2021), it is worth considering compensation for fans for providing the factor of production (Follert et al. 2020). Based on our findings in this present study, we even take a step further and argue that a discussion regarding compensation of fans after sporting success (e.g., winning the championship) is plausible and worth considering.

That fans also hold a majority stake in a football club is not unheard of. For example, the *Exeter Supporter's Trust* holds a majority stake in *Exeter City FC,* which plays in the English League Two (4th division). Although the supporters achieve a consumption benefit from that football match as it is, there is already reciprocity here in that they pay the admission price for this. The bargaining position of the fans is certainly strengthened by the empirical data provided by the studies that we found in our review, so that they could possibly demand greater influence on strategic and operational club decisions. However, it must be considered that fans have strongly limited alternatives for time allocation, provided that they want to spend their free time-consuming football. It is easy to see that a fan of FC Bayern Munich will not switch to Borussia Dortmund if “his” or “her” club denies him or her recognition. Thus, due to their preference structure, fans suffer considerable utility losses when choosing "migration" (Hirschman 1970). Therefore it can be concluded that the stronger the bond between club and fan, the harder the exit will be, since the alternative provides only a comparatively small benefit. With respect to the differentiation provided by Giulianotti (2002), a spectator who is classified as *hot* and *traditional* will almost never switch to another club. This does not mean, of course, that the clubs can act without paying any attention to the fans. Rather, it seems necessary for clubs to produce a minimum level of sporting success in order to keep fans in line in the medium and long term, which in turn has corresponding implications for sports management (signing of players, ticket pricing, etc.).

Besides that, the market power of the fans must not be overestimated from a different perspective: The market power of the fans essentially depends on their level of organization. If the fans can confront the clubs as a closed cartel, they are certainly able to assert their interests. Social media enable such an organization and reduce the corresponding communication costs, but on the one hand, the number of fans is very high and on the other hand, their interests are often very differentiated. Thus, an appearance of the fans as a closed cartel seems rather unlikely, which means that the price-setting scope of the clubs should remain comparatively high.

However, the role of fans can also be used for strategic purposes. It can be interesting for the organizer of national as well as international competitions to choose the venue and/or matchdays (Goller and Krumer 2020) in such a way that the role of the fans is marginalized, and that a “bias-free athletic performance” of the teams dominates the result of the game. At the same time, it should be noted that this can of course also have effects on the other sub-markets such as the market for sponsoring, TV broadcasting rights and merchandising.

It could also be interesting for the visiting team to purchase tickets for away games and distribute them free of charge to their own fans, in the hope that this subsidy will increase the number of their own fans in the away game and thus at least partially eliminate the home advantage.

### Implications for further research

In line with the classical conceptual framework for research on home advantage (Courneya and Carron 1992), we found strong evidence for the crowd as a crucial game location factor. In our systematic literature review the effect of the missing supporters was not only evident with respect to performance and outcome measures (i.e., win ratio, points, goals) in the majority of the studies, but also in measures more directly related to the critical behavioral states of players and referees, such as match dominance (i.e., shots, shots on target), fouls, and awarded cards (Dilger and Vischer 2020; Sors et al. 2020).

From a behavioral science perspective, the consequent relevant question would be about the underlying psychological states of the players and referees. At the moment, the relationship between behavioral and psychological states can only be indirectly inferred from the present data (Webb 2020). Anecdotal evidence from interviews with players (Guardian Football 2020; Hamilton 2021) and referees (UEFA.tv 2020; ZDFsport 2020), however, indicates a substantial impact of the missing supporters on the subjective experience of these sports professionals.

Besides qualitative interviews and self-report questionnaires, a promising approach was recently put forward by Leitner and Richlan (2021b). Their “Analysis System for Emotional Behavior in Football “ (ASEB-F) is a video-based categorical analysis system of nonverbal behavior during football matches. It assumes that emotions can be observed and described as an organized psychophysiological reaction to specific events in the environment, rising to overt actions and leading to human (nonverbal) behavior. The ASEB-F was used to video analyze the behavior of players and officials in 20 games of FC Red Bull Salzburg before and during the COVID-19 pandemic. There were about 20% fewer emotional situations in matches without supporters compared to matches with supporters. In addition, referees were markedly less actively involved in these emotional situations. The results indicate that the absence of supporters has a substantial influence on the experience and behavior of players and officials alike (Link and Anzer 2021).

Possibly related to the psychological effects is the question of whether there are particularly home strong or home weak teams, and, if yes, what the underlying mechanisms are. In addition, our systematic literature review revealed studies which reported differences in the effect of the missing supporters on the home advantage between leagues within countries (e.g., first vs. second divisions) and differences between leagues across countries (e.g., German Bundesliga vs. English Premier League). Therefore, not only psychological but also socio-cultural explanations for the effects in question have to be taken into account (e.g., Sánchez and Lavín, 2021).

Apart from football, there is of course the question of how home field advantage changes in other sports. If one follows the model of Courneya and Carron (1992), then a large number of other sports would have to be affected by the loss of the audience. In order to draw a complete picture of the actual impact of the COVID-19 pandemic on home field advantage in general, studies are needed that are not limited to individual sports but combine as many different disciplines as possible.

In summary, pending questions for future research on the home advantage in football concern (among others) are: (a) the psychological basis of the behavioral effects, (b) differences between teams within leagues, (c) differences between leagues within countries, (d) differences between leagues across countries, and (e) the effects of partial attendance (i.e., only a limited number of—primarily—home fans allowed in the stadiums as a measure of normalization after the COVID-19 pandemic) on the home advantage.

### Limitations

When analyzing the data and interpreting the conclusions of the individual studies, the question of a potential publication bias (sometimes also referred to as “file-drawer problem”) came up when looking at the results of our review. Our analyses show that none of the 26—in some cases significantly different—studies included, conclude an increase in home advantage during ghost games. Likewise, the strong clustering in categories 6 & 7 (decreased & strongly decreased home advantage) of the "Conclusion score" is striking (69.2% of all studies). In a similar vein, only 6 of 26 studies concluded that the home advantage did not change significantly as a result of the ghost games in European football. In the light of these findings, it is quite possible that the ghost games might indeed have brought a significant reduction in home advantage. Nevertheless, the possibility must be considered that results from other studies—that were not published due to “scientifically unpopular results”—would potentially weaken the effect of significantly reduced home advantage during the COVID-19 related ghost games in our review study.

Another key issue to consider when addressing the question of the impact of ghost games on home advantage is how to operationalize home advantage. In this context, different approaches and various constructs can be found in the scientific literature (e.g., Leitner and Richan, 2021a; Matos et al. 2020). While some studies choose the win-ratio or gained/lost points to represent home advantage, other study authors decided to rather analyze the distribution of yellow and red cards to the home and away teams. There is also the empirical approach to analyze match specific aspects, such as match dominance (e.g., characterised by ball possession, shots on target or successful tackles) or other sport-performance related characteristics. Especially in a review study, this divergent approach creates potential problems in an inferential statement. However, our present work does not attempt to make a judgement on these different approaches. Rather, we argue that these circumstances need to be taken into account in a next evaluation, but that at the same time it does justice to a broad overall picture of the influence of ghost games on home advantage in professional football.

We decided to classify the literature using a metric (Spectrum score) to provide the scientific community with a tool to evaluate the basis of the included studies’ results. However, we would like to emphasize that this is an *approximation*. Thus, we explicitly state that this value score is not intended to criticize the authors themselves behind the studies, nor the scientific quality of the respective publications. Rather, our aim in developing the "Spectrum score" was to provide an easy-to-understand measure of the breadth, depth, and number of factors included. We therefore decided to rename the score, originally called "Quality score" (in the first draft of this manuscript), to "Spectrum score", because it reflects the underlying idea more adequately than the original name.

## Conclusion

The COVID-19 pandemic is one of the most drastic crises since the last world war affecting all areas of society, including sports in general and professional football in particular. This gives rise to numerous questions that are of both practical and scientific interest. In addition to economic issues, such as the viability of clubs without spectator revenues, the changed stadium atmosphere is particularly striking. Early in the pandemic, there was anecdotal evidence that the behavior of players changed, e.g., toward referees, and recent studies indicate that the missing crowd indeed has an impact on the (nonverbal) behavior of players, staff and referees (Leitner and Richlan 2021b). Since games without spectators were played in almost all European leagues from mid-2020 at the latest, the situation can be compared to a natural experiment. Thus, we present a systematic literature review—based on 21 empirical studies—focusing on the importance of football fans, who face the restrictions in the pandemic of so-called “ghost games”. Apart from reviewing the main results of the papers, we provide a detailed analysis of study characteristics. For this purpose, we developed a metric that can help to approximate the studies’ width, depth and number of included factors. Our results suggest that home advantage declined in the wake of the COVID-19 ghost games, and that this can be attributed primarily to reduced referee bias and a lack of emotional support from the ranks. We conclude that fans play a significant role in the success of their own team and argue that a discussion about compensation for fans—for example after victories in championship or cup competitions—is legitimate.

## Supplementary Information

Below is the link to the electronic supplementary material.

Supplementary file1 (PDF 2221 KB)

## Data Availability

The data for this study is based on (open-access) studies and publications. See work marked with a hashtag (#) in the References section.

## Footnotes

1 A more recent summary of previous empirical research results on the home advantage can be found in Strauß and MacMahon (2020) and in Peeters and van Ours (2020).

2 It could be argued that due to the numerous infection control regulations, travel and preparation for the game may be different and affect the teams. This is certainly true for both home and away teams, who may have to gather at a hotel for quarantine several days before a match.

3 We follow Fisch and Block (2018) in their understanding of a systematic literature review in management research that this term “refers to all literature reviews that follow a systematic, transparent, and reproducible process for identifying academic literature about a clearly defined topic or research question”.

4 A related methodology is the bibliometric analysis in the form of content analysis, e.g., Mondello and Pedersen (2003).

5 E.g., comments like Bandyopadhyay (2021) were excluded because they do not involve an empirical investigation of the phenomenon. The same applies to newspaper articles that are regularly only descriptive or commentary.

6 For example, the article by Wicke and Bolognesi (2020), which appeared in the search but dealt with the discussion of the pandemic on Twitter, was excluded.

7 In the course of the time between initial database search and manuscript’s review process, 10 additional studies were added to the systematic literature review: Correia-Oliveira 2021; Cross 2020; Cueva 2020; Endrich 2020; Ferraresi 2020; Ferraresi 2021; Krawczyk 2020; Link 2021; Ramchandani 2021; Rovetta 2021.
