# Supplementary material for: The cauldron has cooled down: a systematic literature review on home advantage in football during the COVID-19 pandemic from a socio-economic and psychological perspective
Source: Manag Rev Q. 2022 Jan 4;73(2):605–33. doi: 10.1007/s11301-021-00254-5 (PMC8724651; doi:10.1007/s11301-021-00254-5)
Supplement: Supplementary file 1 — Supplementary file1 (PDF 2221 KB) [file 11301_2021_254_MOESM1_ESM.pdf]

Included studies and individual characteristics (sorted by year and authors)									
Year / Authors	Journal; Platform / Status	Title	Number of countries	Number of analyzed leagues	Analyzed leagues and (Tiers)	League comparison	Number of analyzed seasons	Analyzed factors	Results evaluation
2020 / Cross J; Uhrig R	SSRN / not peer-reviewed yet	Do Fans Impact Sports Outcomes? A COVID-19 Natural Experiment	4	4	England (1); Germany (1); Italy (1); Spain (1)	Across	11 (2009 - 2019)	Win ratio; Goals; Expected goals	No
2020 / Cueva C	Econstor / not peer-reviewed yet	Animal Spirits in the Beautiful Game	30	41	England (1, 2, 3, 4, 5); Spain (1, 2); Italy (1, 2); France (1, 2); Germany (1, 2); Turkey (1); Portugal (1); Belgium (1); Greece (1); Scotland (1); Brazil (1); USA (1); Mexico (1); Japan (1); Poland (1); Romania (1); Sweden (1); Norway (1); Russia (1); Denmark (1); Finland (1); Ireland (1); China (1); South Korea (1); Austria (1); Switzerland (1); Bulgaria (1); Hungary (1); Netherlands (1); Scotland (1, 2, 3); Argentina (1)	Across	27 (1994 - 2020)	Win ratio; Fouls; Cards	Yes

2020 / Dilger A; Vischer L	Econstor / not peer-reviewed yet	No home bias in ghost games	1	1	Germany (1)	N/A	1 (2019)	Win ratio; points; goals; market value; extra time; shots; distance covered; passes; possession; tackles; fouls; yellow cards; red cards	Yes
2020 / Endrich M; Gesche T	Economics Letters / peer-reviewed	Home-bias in referee decisions: Evidence from "Ghost Matches" during the Covid19-Pandemic	1	2	Germany (1, 2)	N/A	1 (2019)	Fouls; Cards	No
2020 / Ferraresi M; Gucciardi G	Econpapers / not peer-reviewed yet	Team performance and audience: experimental evidence from the football sector	5	5	France (1); Germany (1); Italy (1); Spain (1); England (1)	Across	1 (2019)	Win ratio; Points	No
2020 / Krawczyk M; Strawinski P	Econpapers / not peer-reviewed yet	Home advantage revisited. Did COVID level the playing fields?	4	4	England (1); Germany (1); Italy (1); Spain (1)	Individual	3 (2017 - 2019)	Win ratio; Points, Goals	Yes

2020 / Matos R; Monteiro D; Antunes R; Mendes D; Botas J; Clemente J; Amaro N	International Journal of Environmental Research and Public Health / peer-reviewed	Home-Advantage during COVID-19: An Analysis in Portuguese Football League	1	1	Portugal (1)	N/A	4 (2016 - 2019)	Points	Yes
2020 / Sors F; Grassi M; Agostini T; Murgia M	European Journal of Sport Science / peer-reviewed	The sound of silence in association football: Home advantage and referee bias decrease in matches played without spectators	4	8	Germany (1, 2); England (1, 2); Spain (1, 2); Italy (1, 2)	Across	4 (2016 - 2019)	Win ratio; Points; Goals; Ball possession; Shots; Shots on target; Corner kicks; Fouls; Cards; Penalty kicks; Extra time	Yes
2020 / Tilp M; Thaller S	frontiers in Sports and Active Living / peer-reviewed	Covid-19 Has Turned Home Advantage Into Home Disadvantage in the German Soccer Bundesliga	1	1	Germany (1)	N/A	2 (2018 - 2019)	Win ratio; Fouls; Cards; Penalty kicks	Yes
2021 / Almeida CH; Leite WS	Biology of Sport / peer-reviewed	Professional football in times of COVID-19: did the home advantage effect disappear in European domestic leagues?	5	5	Germany (1); Spain (1); England (1); Portugal (1); Italy (1)	Across & Individual	1 (2019)	Points; Goals; Shots; Possession; Passes; Aerial Duels; Tackles; Cards	No

2021 / Benz LS; Lopez MJ	AstA Advances in Statistical Analysis / peer-reviewed	Estimating the change in soccer's home advantage during the Covid-19 pandemic using bivariate Poisson regression	13	17	Germany (1, 2); Denmark (1); Austria (1); Portugal (1); Greece (1); Spain (1, 2); Turkey (1); Sweden (1); Norway (1); England (1, 2); Italy (1, 2); Switzerland (1); Russia (1)	Individual	5 (2015 - 2019)	Goals; Cards	No
2021 / Bryson A; Dolton P; Reade JJ; Schreyer D; Singleton C	Economics Letters / peer-reviewed	Causal effects of an absent crowd on performances and refereeing decisions during Covid-19	17	23	Albania (1); Australia (1); Austria (1, 2); Costa Rica (1); Denmark (1); England (1, 2); Germany (1, 2, 3); Greece (1); Hungary (1); Italy (1, 2); Poland (1); Portugal (1); Romania (1); Serbia (1); Slovenia (1); Spain (1, 2); Ukraine (1)	Across & Individual	1 (2019)	Win ratio; Attendance; Goals, Cards	No
2021 / Correia-Oliveira; Andrade-Souza	International Journal of Sport and Exercise Psychology / peer-reviewed	Home advantage in soccer after the break due to COVID-19 pandemic: does crowd support matter?	4	7	Germany (1, 2); Spain (1, 2); England (1, 2); Italy (1)	Individual	4 (2016 - 2019)	Win ratio; Points; Goals; Attendance	Yes

2021 / Ferraresi M; Gucciardi G	Economics Letters / peer-reviewed	Who chokes on a penalty kick? Social environment and individual performance during Covid-19 times	5	5	France (1); Germany (1); Italy (1); Spain (1); England (1)	Across	1 (2019)	Scored and unscored penalties	No
2021 / Fischer K; Haucap J	Journal of Sports Economics / peer-reviewed	Does Crowd Support Drive the Home Advantage in Professional Soccer? Evidence from German Ghost Games during the COVID-19 Pandemic	1	3	Germany (1, 2, 3)	Individual	4 (2017 - 2019)	Win ratio; points; player value; table position; pause (from last match); shape (points earned in last three matches); travel distance; altitude; new coach; matchday; daytime; derby; stadium track, share standing places, capacity; yellow cards, fouls; shots; corners	Yes
2021 / Hill Y; Yperen N	frontiers in Psychology / peer-reviewed	Losing the Home Field Advantage When Playing Behind Closed Doors During COVID-19: Change or Chance?	4	4	Germany (1); Spain (1); England (1); Italy (1)	Across & Individual	5 (2015 - 2019)	Points; goals; shots; possession; fouls; cards	No

2021 / Konaka E	arXiv / not peer-reviewed yet	Home advantage of European major football leagues under COVID-19 pandemic	4	4	England (1); Germany (1); Italy (1); Spain (1)	Across & Individual	10 (2010 - 2019)	Win ratio; Goals	No
2021 / Leitner MC; Richlan F	frontiers in Sports and Active Living / peer-reviewed	No Fans - No Pressure: Referees in Professional Football During the COVID-19 Pandemic	8	8	Spain (1); England (1); Germany (1); Italy (1); Russia (1); Turkey (1); Austria (1); Czech Republic (1)	Across	3 (2018 - 2019)	Win ratio; Fouls; Cards; Cards in the course of the game	Yes
2021 / Link; D; Anzer G	International Journal of Sports Medicine / peer-reviewed	How the COVID-19 Pandemic has Changed the Game of Soccer	1	2	Germany (1, 2)	N/A	3 (2017 - 2019)	Win ratio; Goals; Cards; Fouls; Shots; Expected Goals; Ball Possession; Passes; Outplayed Opponents; Pressure; Duels; Running Activity; Contact times	No

2021 / McCarrick D; Bilalic M; Neave N; Wolfson S	Psychology of Sport & Exercise / peer-reviewed	Home advantage during the Covid-19 pandemic: Analyses of European football leagues	11	15	England (1, 2); Germany (1, 2); Spain (1, 2); Italy (1, 2); Portugal (1); Greece (1); Turkey (1); Austria (1); Denmark (1); Russia (1); Switzerland (1)	Across	2 (2019)	Points; Goals, Corners, Shots, Shots on Target, Fouls, Cards	Yes
2021 / Ramchandani G; Millar R	Journal of Global Sport Management / peer-reviewed	Investigating the "Twelfth Man" Effect in Five European Domestic Football Leagues: A COVID-19 Induced Natural Experiment	5	5	England (1); Germany (1); Italy (1); Portugal (1); Spain (1)	Across & Individual	2 (2018 - 2019)	Win ratio; Points	No
2021 / Rovetta A; Abate A	Cureus / peer-reviewed	The Impact of Cheering on Sports Performance: Comparison of Serie A Statistics Before and During COVID-19	1	1	Italy (1)	N/A	8 (2013 - 2020)	Points; Passes; Fouls, Penalties, Cards	Yes

2021 / Sánchez AJ; Lavín JM	Soccer & Society / peer-reviewed	Home advantage in European soccer without crowd	5	8	Germany (1, 2); Spain (1, 2); Italy (1, 2); England (1); Austria (1)	Across & Individual	1 (2019)	Points; Goals; Table position; Stadium performance; Number of foreigners; Players' experience; Cards; Budget; Average attendance	Yes
2021 / Santana H; Bettega O; Dellagrana R	Science and Medicine in Football / peer-reviewed	An analysis of Bundesliga matches before and after social distancing by COVID-19	1	1	Germany (1)	N/A	1 (2019)	Win ratio; Goals; Goal attempts; Possession; Passes; Passes accuracy; Tackles; Corners; Offside; Fouls; Team distance covered; Number of sprints	No
2021 / Scoppa V	Journal of Economic Psychology / peer-reviewed	Social pressure in the stadiums: Do agents change behavior without crowd support?	5	9	Germany (1, 2); England (1, 2); Spain (1, 2); Italy (1, 2); Portugal (1)	Across	10 (2010 - 2019)	Points; Goals; Shots; Shots on target; Corners; Fouls; Cards; Penalties; Rest	No

2021 / Wunderlich F; Weigelt M; Rein R; Memmert D	PLOS ONE / peer-reviewed	How does spectator presence affect football? Home advantage remains in European top-class football matches played without spectators during the COVID-19 pandemic	6	11	Spain (1, 2); England (1, 2); Italy (1, 2); Germany (1, 2, 9); Portugal (1); Turkey (1)	Across	10 (2010 - 2019)	Fouls; Cards; Shots; Shots on target; Goals; Betting odds; Points	Yes

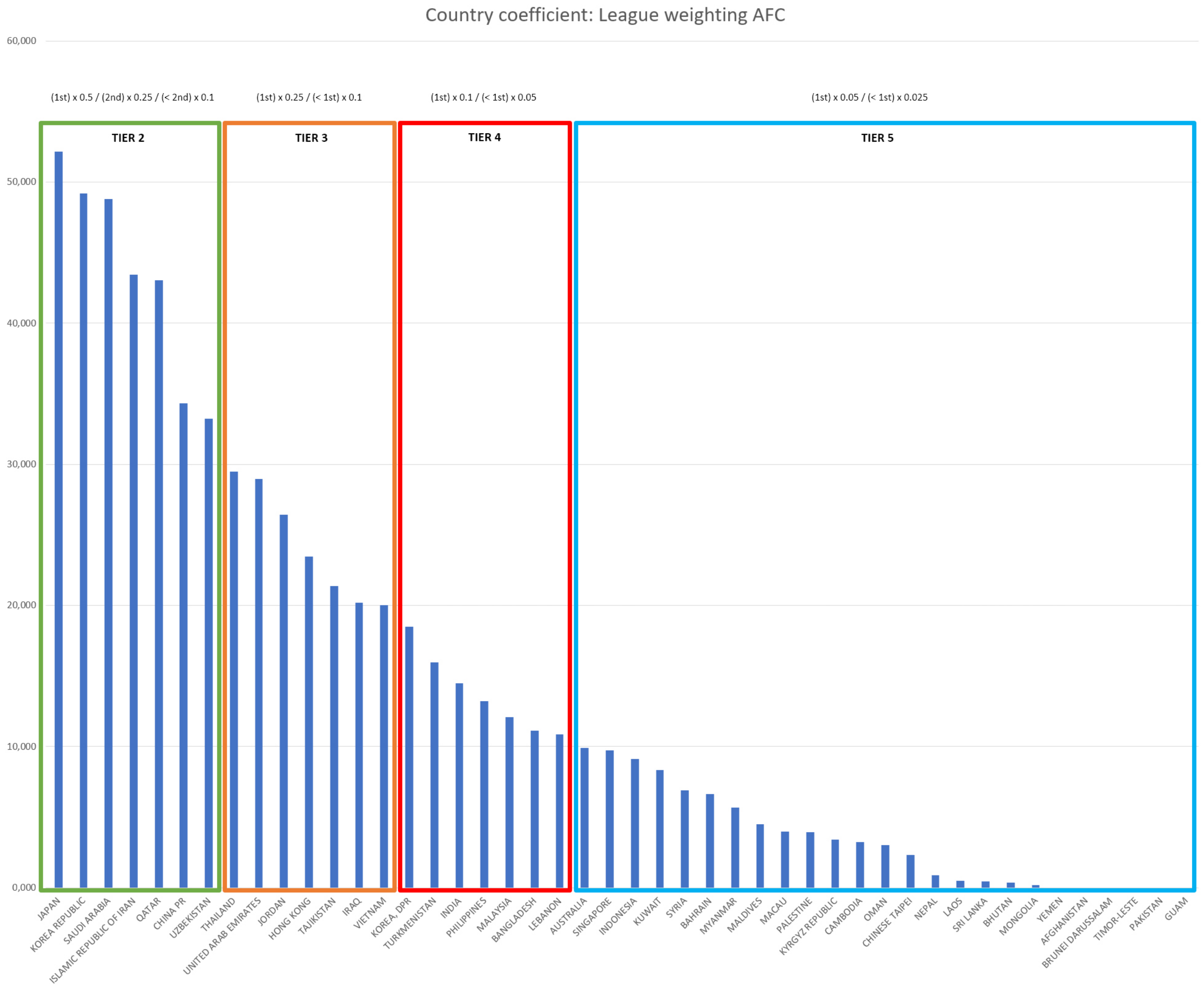
Country coefficient: League weighting AFC
60,000
50,000
40,000
30,000
20,000
10,000
0,000
(1st) x 0.5 / (2nd) x 0.25 / (< 2nd) x 0.1
(1st) x 0.25 / (< 1st) x 0.1
(1st) x 0.1 / (< 1st) x 0.05
(1st) x 0.05 / (< 1st) x 0.025
TIER 2
TIER 3
TIER 4
TIER 5
JAPAN
KOREA REPUBLIC
SAUDI ARABIA
ISLAMIC REPUBLIC OF IRAN
QATAR
CHINA PR
UZBEKISTAN
THAILAND
UNITED ARAB EMIRATES
JORDAN
HONG KONG
TAJIKISTAN
IRAQ
VIETNAM
KOREA, DPR
TURKMENISTAN
INDIA
PHILIPPINES
MALAYSIA
BANGLADESH
LEBANON
AUSTRALIA
SINGAPORE
INDONESIA
KUWAIT
SYRIA
BAHRAIN
MYANMAR
MALDIVES
MACAU
PALESTINE
KYRGYZ REPUBLIC
CAMBODIA
OMAN
CHINESE TAIPEI
NEPAL
LAOS
SRI LANKA
BHUTAN
MONGOLIA
YEMEN
AFGHANISTAN
BRUNEI DARUSSALAM
TIMOR-LESTE
PAKISTAN
GUAM

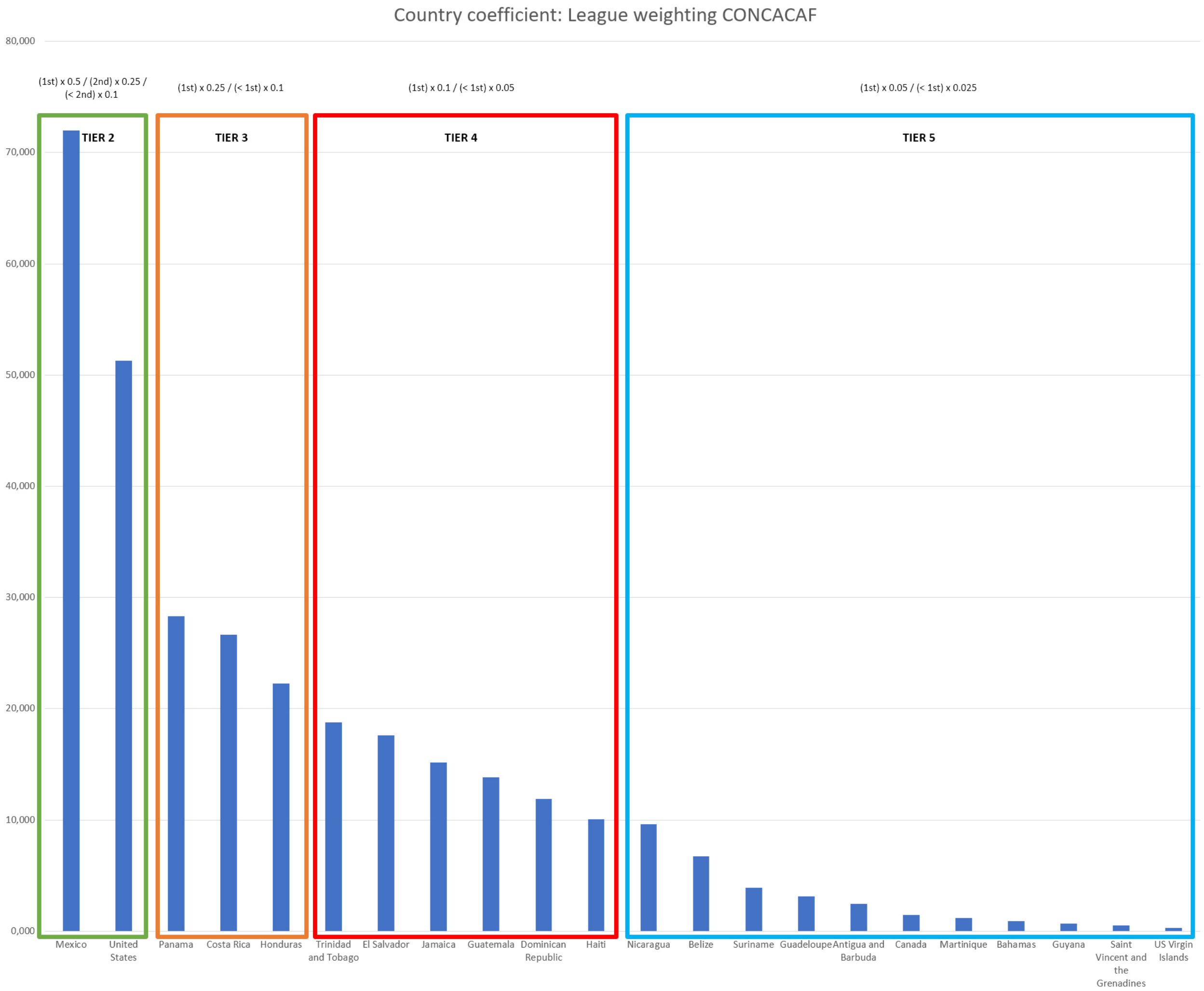

Country coefficient: League weighting CONCACAF
80,000
70,000
60,000
50,000
40,000
30,000
20,000
10,000
0,000
(1st) x 0.5 / (2nd) x 0.25 / (< 2nd) x 0.1
(1st) x 0.25 / (< 1st) x 0.1
(1st) x 0.1 / (< 1st) x 0.05
(1st) x 0.05 / (< 1st) x 0.025
TIER 2
TIER 3
TIER 4
TIER 5
Mexico
United States
Panama
Costa Rica
Honduras
Trinidad and Tobago
El Salvador
Jamaica
Guatemala
Dominican Republic
Haiti
Nicaragua
Belize
Suriname
Guadeloupe
Antigua and Barbuda
Canada
Martinique
Bahamas
Guyana
Saint Vincent and the Grenadines
US Virgin Islands

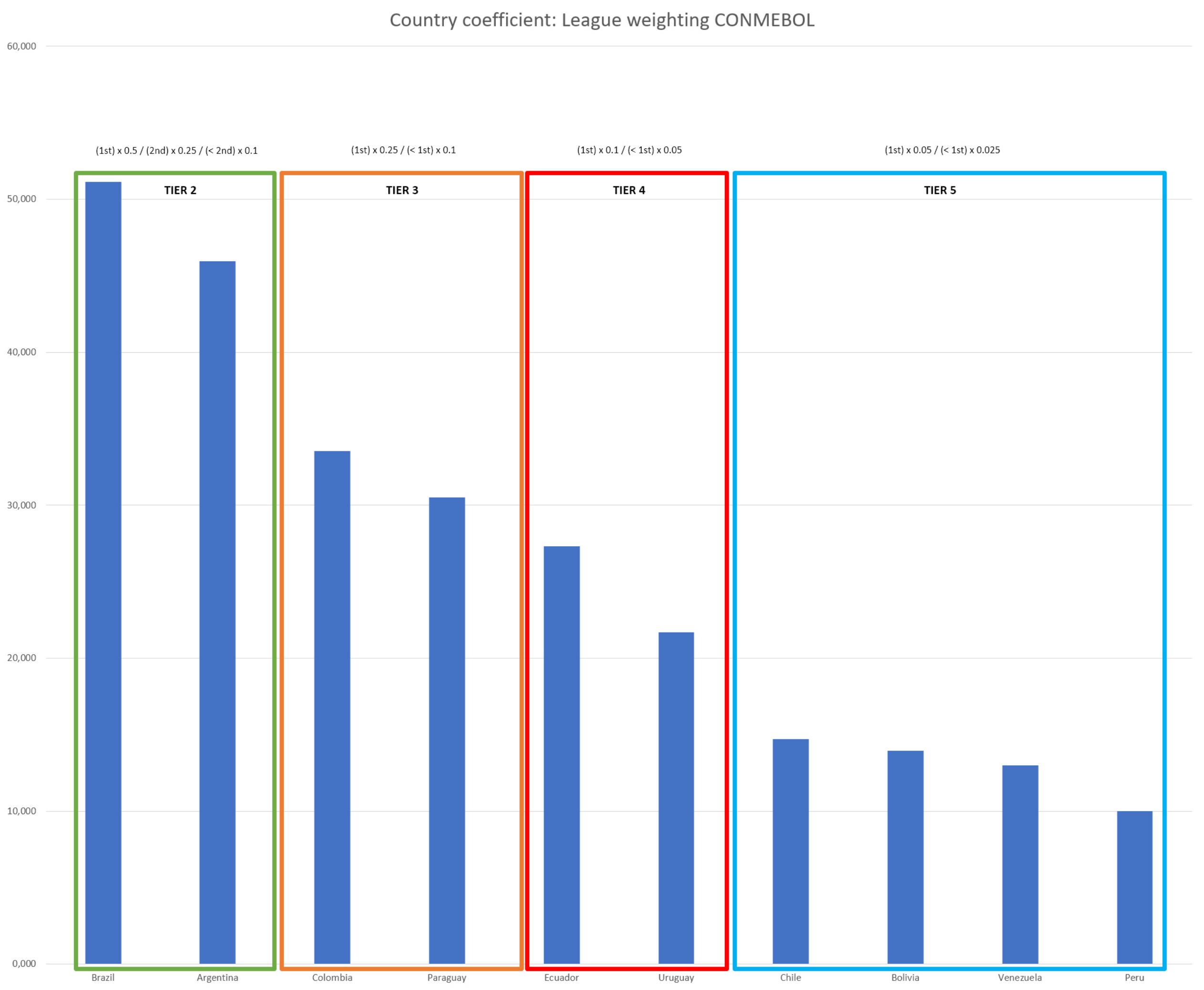

Country coefficient: League weighting CONMEBOL
60,000
50,000
40,000
30,000
20,000
10,000
0,000
(1st) x 0.5 / (2nd) x 0.25 / (< 2nd) x 0.1
(1st) x 0.25 / (< 1st) x 0.1
(1st) x 0.1 / (< 1st) x 0.05
(1st) x 0.05 / (< 1st) x 0.025
TIER 2
TIER 3
TIER 4
TIER 5
Brazil
Argentina
Colombia
Paraguay
Ecuador
Uruguay
Chile
Bolivia
Venezuela
Peru

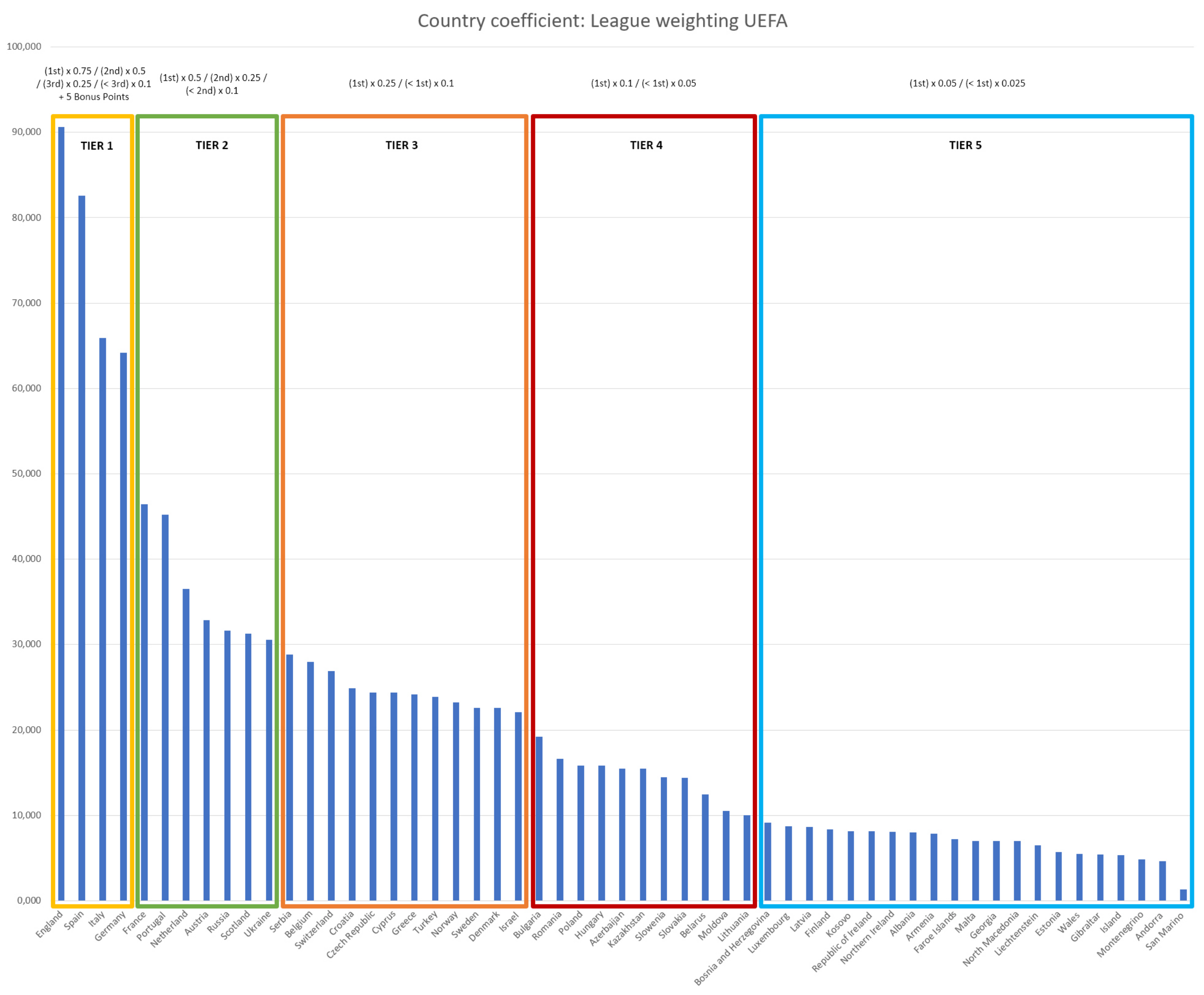
Country coefficient: League weighting UEFA
(1st) x 0.75 / (2nd) x 0.5 / (3rd) x 0.25 / (< 3rd) x 0.1 + 5 Bonus Points
(1st) x 0.5 / (2nd) x 0.25 / (< 2nd) x 0.1
(1st) x 0.25 / (< 1st) x 0.1
(1st) x 0.1 / (< 1st) x 0.05
(1st) x 0.05 / (< 1st) x 0.025
TIER 1
TIER 2
TIER 3
TIER 4
TIER 5
100,000
90,000
80,000
70,000
60,000
50,000
40,000
30,000
20,000
10,000
0,000
England
Spain
Italy
Germany
France
Portugal
Netherland
Austria
Russia
Scotland
Ukraine
Serbia
Belgium
Switzerland
Croatia
Czech Republic
Cyprus
Greece
Turkey
Norway
Sweden
Denmark
Israel
Bulgaria
Romania
Poland
Hungary
Azerbaijan
Kazakhstan
Slowenia
Slovakia
Belarus
Moldova
Lithuania
Bosnia and Herzegovina
Luxembourg
Latvia
Finland
Kosovo
Republic of Ireland
Northern Ireland
Albania
Armenia
Faroe Islands
Malta
Georgia
North Macedonia
Liechtenstein
Estonia
Wales
Gibraltar
Island
Montenegrino
Andorra
San Marino
